# Is Hypovitaminosis D a Risk Factor for Heart Failure?

**DOI:** 10.3390/life13020372

**Published:** 2023-01-29

**Authors:** Asmaa Carla Hagău, Amalia Pușcaș, Rodica Togănel, Iolanda Muntean

**Affiliations:** 1 Doctoral School of Medicine and Pharmacy, I.O.S.U.D., George Emil Palade University of Medicine, Pharmacy, Science, and Technology of Targu Mures, Emergency Institute for Cardiovascular Diseases and Transplantation of Târgu Mureș, 540136 TarguMures, Romania; 2 Department of Biochemistry and Chemistry of Environmental Factors, Faculty of Pharmacy, University of Medicine, Pharmacy, Science and Technology “George Emil Palade” of Targu Mures, 540142 Targu Mures, Romania; 3 Department of Paediatrics, George Emil Palade University of Medicine, Pharmacy, Science, and Technology of Targu Mures, 540142 Targu Mures, Romania; 4 Clinic of Paediatric Cardiology, Emergency Institute for Cardiovascular Diseases and Transplantation of Târgu Mureș, University of Medicine, Pharmacy, Sciences and Technology “George Emil Palade” of Targu Mures, 540142 Târgu Mureș, Romania

**Keywords:** Vitamin D deficiency, cardiovascular, risk factors, heart failure, adults, children

## Abstract

Background: Several studies in recent years have shown the association between vitamin D levels and heart failure. Vitamin D deficiency is related to increased cardiovascular morbidity and mortality, with a higher risk of developing heart failure. In this systematic review, we aimed to assess recent studies that analyzed vitamin D deficiency and heart failure in adult and pediatric populations. (2) Methods: We conducted a systematic search for studies published in the following databases: PubMed and Scopus from January 2012 to October 2022. (3) Results: Most observational studies that were included found a significant association between hypovitaminosis D and heart failure. However, the beneficial role of vitamin D supplementation is still controversial due to the lack of randomized controlled trials. (4) Conclusions: Vitamin D may play an important role as a cardiovascular marker in heart failure patients. More well-designed studies are needed to investigate the relationship between vitamin D and heart failure and to determine if vitamin D supplementation could improve long-term outcomes.

## 1. Introduction

According to the European Society of Cardiology, heart failure (HF) is one of the leading causes of hospitalization in people over the age of 65, with an incidence of 1–2% in the general population and more than 10% in patients above 70 years [1]. In the United States, almost 6 million patients have HF, with an annual mortality rate of over 300.000 deaths [2]. Despite recent advances in pharmacological therapy, cardiovascular diseases remain a major contributor to mortality and morbidity in both pediatric and adult populations. One of the primary reasons for this is that as life expectancy has increased, so has the number of elderly patients with significant comorbidities such as diabetes mellitus or hypertension. Even though multiple risk factors for cardiovascular diseases are known, in recent years, studies have focused on novel risk factors that can predict patient prognosis. Because vitamin D is a modifiable factor, its effects on different systems have received attention in recent years. Numerous studies have linked hypovitaminosis D as a risk factor for cardiovascular diseases such as HF, hypertension, or atrial fibrillation. However, the role of vitamin D in preventing and treating cardiovascular diseases is still debatable because results from observational trials and randomized controlled trials are controversial. Therefore, this review aims to summarize the implications of vitamin D in HF adults and pediatric patients. 

## 2. Materials and Methods

**Figure 1:**
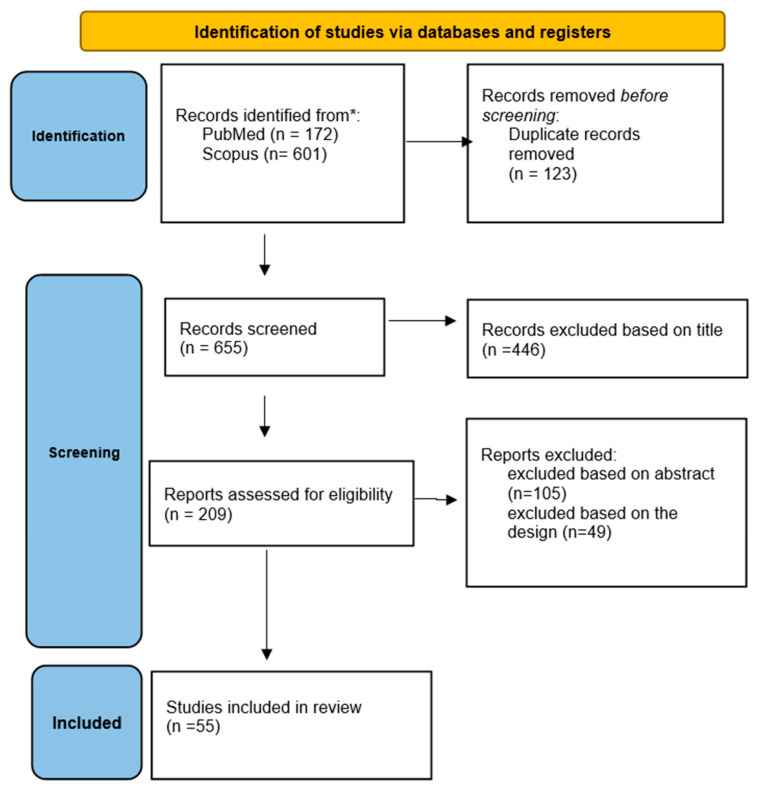
PRISMA 2022 flow diagram.

This paper is a systematic review of articles offered by the scientific literature regarding vitamin D deficiency and supplementation in the context of HF published in the following databases: PubMed and Scopus from January 2012 to October 2022. We included original research studies on human subjects from both adult and pediatric populations, written in English, that summarize the available evidence regarding the role of vitamin D in heart failure. Papers and experimental studies that were not available in English were excluded. Experimental studies were being used only to describe the pathophysiological mechanisms involved. The following keywords were used for the search: “heart failure and vitamin D”, “vitamin D deficiency”, and ”Vitamin D and cardiovascular”. To summarize the review process, we used the Preferred Reporting Item for Systematic Review and Meta-analysis (PRISMA) 2020 guidelines, which are shown in Figure 1. After removing duplicates with EndNote 9, screening of title and abstract, application of eligibility criteria, and quality appraisal, articles were retained for systemic review. 

## 3. Mechanism of Action

Vitamin D is considered a steroid hormone that is known for its crucial role in calcium homeostasis and its actions on the musculoskeletal system. 

Vitamin D is usually obtained through either sunlight exposure or dietary intake. The cutaneous synthesis of vitamin D begins in the skin after exposure to ultraviolet light when the precursor molecule 7-dehydrocholesterol is photoconverted to provitamin D3. After this process, vitamin D ingested from the diet and synthesized in the skin requires two hydroxylation processes catalyzed by 1α-Hydroxylase; the first process occurs in the liver, where 25-hydroxyvitamin D (25-OHD) is formed, and the second one in the kidneys, where the active metabolite of vitamin D, 1α,25-dihydroxy vitamin D (1.25-OHD), is formed [3]. 

The biological effects of 1.25-OHD are regulated both directly and indirectly. First and foremost, in vitro studies discovered the presence of 1α,-Hydroxylase in multiple tissues throughout the body, such as intestinal cells, cardiomyocytes, smooth muscle fibers and lymphocytes, implying that the biologically active form of vitamin D can be converted into cells other than hepatic and renal cells [4]. Second, 1.25-OHD binds to the intracellular vitamin D receptors (VDR). Due to its liposoluble molecule, vitamin D has the potential to cross the cellular membrane and bind with VDR in the cytoplasm of the cells. Together, vitamin D and VDR form a complex that translocates into the nucleus and activates the transcription of specific genes responsible for the vitamin D effects, therefore regulating gene expression. Experimental studies have shown that VDR is also present in the cytoplasm of almost all tissues, including cardiomyocytes, vascular smooth cells, fibroblasts and immune cells [5]. Therefore, due to its genomic and non-genomic effects, studies have estimated that vitamin D is capable of controlling almost 3% of the human genome [6]. This might explain the numerous actions of vitamin D regarding other tissues, such as the cardiovascular, endocrine or immune systems. 

Although 1.25-OHD is the biologically active form of the hormone, it is rarely used in clinical practice because its serum concentration can be influenced by other external factors. For example, the production of 1.25-OHD can be influenced by renal diseases, phosphorus and calcium can suppress 1.25-OHD synthesis and parathyroid hormone (PTH) can stimulate its synthesis [7]. Therefore, in order to determine vitamin D deficiency, the pre-hormone 25-OHD is usually used in clinical practice due to its relative stability, an almost 1000 times higher concentration in the plasma and a longer half-time of approximately 3 weeks [8]. However, it is important to mention that 25-OHD levels may not accurately reflect vitamin D status in patients with different diseases and physiological factors that can affect only the active metabolite. For example, to limit research bias in clinical practice, in some patients that associate bone mineral disease or renal diseases, it is recommended to use the active metabolic form to evaluate vitamin D status. Based on 25-OHD levels, there are many definitions regarding vitamin D normal concentrations. However, there is still a lack of consensus among specialized societies regarding vitamin D status levels. According to the American Society of Pediatrics and Medicine Institute, vitamin D deficiency is considered when 25-OHD levels are below 20 ng/mL, vitamin D insufficiency is considered when 25-OHD is between 20–29 ng/mL and optimal level of vitamin D when 25-OHD levels are above 30 ng/mL [9]. However, the Endocrinology Society considers vitamin D insufficiency when 25-OHD levels are below 30 ng/mL and optimal when 25-OHD levels are above 30 ng/mL (>75 nmol/L) [10]. In addition, the recommendation of optimal vitamin D supplementation is still debatable, ranging between 600–2000 international units (IU) [11,12,13]. Despite the novel studies involving vitamin D in cardiovascular diseases, current recommendations for vitamin D supplementation are still based on the vitamin D levels needed to prevent rickets in pediatric patients and osteomalacia in the adult population.

Across the globe, according to World Health Organization, approximately a million people suffer from vitamin D deficiency. As stated above, clinicians use 25-OHD to determine vitamin D deficiency, so these numbers may not accurately reflect the active metabolite 1.25-OHD status in the general population. To begin, some important factors that can affect vitamin D production include insufficient sunlight exposure, changing seasons, increased sunscreen use, aging and an unbalanced dietary intake. Furthermore, skin pigmentation can interfere with vitamin D production because higher levels of melanin in the epidermis reduce ultraviolet light penetration, making people with darker skin more prone to vitamin D deficiency [14]. In the pediatric population, breastfeeding infants are more predisposed to have vitamin D deficiency due to low concentrations of vitamin D in breast milk [15]. Epidemiological studies have revealed that nearly 80% of patients with HF have hypovitaminosis D. In both clinical and experimental studies, vitamin D levels have been linked to chronic HF [16].

## 4. Vitamin D and Heart Failure 

Heart failure is a complex pathophysiological syndrome characterized by typical signs and symptoms in which the ability of the heart to supply blood is impaired due to a structural or functional cardiac pathology [1]. Because it is the final step in most cardiovascular diseases, it is regarded as a major health burden worldwide. 

Some proposed hypotheses could explain the association between vitamin D and HF. Low vitamin D levels can activate several HF pathogenesis pathways, including cardiac remodeling, inflammation, calcium-modulating effects and renin-angiotensin-aldosterone system (RAAS). In addition, hypovitaminosis D can contribute to the progression of HF. 

To begin with, experimental studies have shown that vitamin D has local effects on cardiomyocytes via local conversion controlled by 1α-Hydroxylase. The action of 1.25-OHD at voltage-gated calcium channels in cardiomyocytes causes a rapid influx of calcium in cardiac cells. First, it is known that calcium plays a key role in cardiac contractility, with a *positive inotropic effect* [17]. Second, the binding between 1.25-OHD to VDR modulates the indirect effects of 1.25-OHD on cardiomyocytes by altering RAAS. Experimental studies have shown the critical role played by vitamin D as a potent regulator of renin biosynthesis. In vitro, 1.25-OHD binding to VDR has the potential to suppress renin gene transcription regardless of calcium levels. Increased renin and angiotensin II levels have also been reported in animal models using VDR-null mice. As a result, in VDR-null mice, renin and angiotensin II overproduction was linked to the development of cardiac hypertrophy as well as increased water and sodium absorption, resulting in hypertension [18,19]. Third, experimental studies have also described the effect of vitamin D on extracellular matrix turnover, with the activation of tissue inhibitors of matrix-metalloproteinases, implying an anti-fibrotic and cardioprotective effect of vitamin D against the cardiac remodeling process [5]. These findings were also supported by in vitro studies and histological studies on explanted hearts, which found a significant association between low 25-OHD levels and fibrosis [20,21]. In addition, chronic HF is characterized by persistent systemic inflammation and an imbalance between the production of oxygen free radicals and antioxidants, resulting in oxidative stress. Accumulation of free oxygen radicals increases pro-inflammatory cytokines, such as TNF-alpha, IL-1 and IL-6, which causes cardiomyocyte death and intensifies cardiac dysfunction. The maladaptive process of replacing necrotic myocytes results also in cardiac fibrosis [22,23]. In vitro studies have linked hypovitaminosis D with elevated TNF-α levels and decreased IL-10 concentrations, suggesting an immunomodulatory effect and anti-inflammatory response of vitamin D. Furthermore, it is known that hypovitaminosis D causes hypocalcemia that stimulates parathyroid hormone secretion, another risk factor currently investigated for its involvement in cardiovascular diseases [24,25,26]. Recent studies also support these findings (Table 1).

It is important to mention that dilated cardiomyopathy (DCM) is considered one of the leading causes of advanced HF, making DCM the most common indication for adult and pediatric heart transplants [34]. In the adult population, observational studies found a significant association between 25-OHD levels and advanced HF. Karoli et al. found that 25-OHD serum levels were significantly lower in patients with dilated cardiomyopathy (DCM) and advanced HF compared to the control group (*p* = 0.001). Furthermore, the researchers discovered a significant negative correlation between 25-OHD levels, NT-proBNP (*r* = −0.32; *p* = 0.02) and left ventricle (LV) systolic (*r* = −0.38; *p* = 0.01) and diastolic dimensions (*r* = −0.35; *p* = 0.01) [35]. Similar results were observed in other scientific studies [36,37]. In contrast, Pandit et al. described no association between vitamin D deficiency and advanced HF, but hypovitaminosis D was found to be significantly related to interventricular septum dimensions and LV mass index [38]. However, due to the observational study designs, it is still unclear if vitamin D deficiency is directly involved in DCM and HF pathogenesis or if it is secondary to limited functional capacity and lack of sunlight exposure. Interestingly, in the pediatric population, there are some case reports of infants with DCM associated with hypocalcemia and vitamin D deficiency. After treatment with cardiotonics was initiated and vitamin D and calcium supplementation, myocardial dilatation and dysfunction resolved completely [39,40,41]. In addition, in an observational study with pediatric patients diagnosed with idiopathic DCM, Raafat et al. found a significant negative correlation between vitamin D deficiency and fraction shortening (r = −0.26, *p* = 0.04) and LV end-diastolic diameter (r = −0.37, *p* = 0.01) [42]. These reports suggest that optimal levels of calcium and vitamin D are necessary for myocardial health.

In addition, an association between vitamin D and HF was not only found in patients with advanced HF. Saponaro et al. confirmed the prevalence of lower vitamin D levels in patients with mild/moderate HF [31]. Similar results were reported in a prospective study with a nearly 5-year follow-up period that included patients (no. 780) with early-stage of HF and HF with preserved ejection; lower 25-OHD concentrations at baseline were related to the worse clinical outcome (*p* ≤ 0.002), reduced exercise capacity (*p* ≤ 0.001) and higher NT-proBNP values (*p* ≤ 0.0023). Furthermore, low 25-OHD values were linked to a higher 5-year risk of mortality (*p* = 0.05, hazard ratio (HR) 1.55 [1.00; 2.42]) [32]. Interestingly, while assessing BNP and vitamin D levels in a Japanese population, Otani et al. discovered that despite lower BNP values in patients with preserved ejection fraction compared with those with impaired ejection fraction, both groups had similar impairment of 25-OHD concentration, with 25-OHD levels being a significant independent predictor of death [43].

Surprisingly, similar findings were also seen in healthy patients with hypovitaminosis D. While investigating the relationship between 25-OHD levels and LV function in patients without HF, Fall et al. found significantly lower values of LV end-systolic diameter and better systolic function in patients with higher 25-OHD levels at baseline [44]. In addition, Ameri et al. discovered that 25-OHD was significantly correlated with LV thickness and LV mass index. Furthermore, subjects with lower 25-OHD values were more prone to LV remodeling (odds ratio (OR) 1.24; 95% confidence interval (CI) 0.83–1.85) and LV hypertrophy [45]. Therefore, regardless of the presence of HF, these findings suggest an association between vitamin D status and left ventricular remodeling [38,44,45]. 

Similar results were reported in studies that examined the relationship between vitamin D and HF concerning various comorbidities. In The Ludwigshafen Risk and Cardiovascular Health study conducted on patients with metabolic syndrome (no. 1801), those with optimal levels of vitamin D had an 85% lower risk of sudden cardiac death (HR 0.15 [95% CI 0.04–0.6]) compared to those with severe deficiency of vitamin D (below 25 nmol/L) [46]. It is important to mention that, despite the 7.7-year follow-up, one of the main limitations of this study is that vitamin D status was assessed only at a one-time point, at the beginning of the study, and thus may not reflect vitamin D status in the long term. 

These studies reveal that, despite a low level of vitamin D in the general population, patients with HF have lower levels of vitamin D compared to the general population, regardless of clinical severity or NYHA class. One possible explanation is the lack of sunlight exposure in patients with higher functional NYHA classes. It is well known that HF patients are at risk of having low vitamin D serum levels due to limited effort capacity and insufficient sunlight exposure. However, similar results were found in studies conducted in sunny regions in both adult and pediatric populations, implying that hypovitaminosis D is associated with HF and HF risk factors regardless of sunlight exposure [47,48]. 

It is worth mentioning that comparable results were found in other observational studies that analyzed the biologically active form of vitamin D. Grunson et al. conducted a prospective study including 170 patients with HF who were followed for a median time of 4.1 years and described a significant association between low levels of 1.25-OHD and HF, with 1.25-OHD concentrations being negatively correlated with the severity of HF (*p* < 0.001) [29].

In contrast, a study designed by Kolaszko et al. that examined serum levels of vitamin D and PTH in 127 patients with HF reported that mean PTH serum levels were significantly higher in HF patients with worse NYHA class compared to the non-HF group, but there were no differences in serum levels of vitamin D between the analyzed groups [26]. Wannamethe et al. observed comparable results in a prospective study on 3731 patients who developed HF [25]. However, the Kolaszko study was performed on a small population, with no follow-up measurements, whereas the Wannamethe et al. study examined a population of predominantly white elderly male patients. In conclusion, extrapolating these findings to the general population is difficult.

The immunomodulatory effect of vitamin D in relationship with HF was also analyzed in observational studies. Ma et al. examined the connection between vitamin D levels and pro-inflammatory/anti-inflammatory T cell lymphocytes in patients with HF. This study showed a pro-inflammatory/anti-inflammatory T cell imbalance in HF patients, with anti-inflammatory lymphocyte levels significantly reduced in patients with higher NYHA class compared with the control group. Vitamin D levels were lower in HF patients and positively correlated with anti-inflammatory T cells (𝑟 = 0.3617, *p* = 0.0045). Vitamin D concentrations were found to be inversely related to natriuretic peptide levels (NT-proBNP) (𝑟 = −0.3544, *p* = 0.0044) and higher clinical severity [49]. Afifeh et al. also described an inverse linear relationship between 1.25-OHD and inflammation markers linked to cardiovascular risks, such as C-reactive protein and fibrinogen [50]. Vasques et al. discovered a significant inverse correlation between 25-OHD levels and Il-1, Il-6, Il-8 and TNF-α [51]. Due to the immunomodulatory effect on anti-inflammatory and pro-inflammatory cytokines, these studies suggest that vitamin D supplementation in HF patients may play an important role in preventing HF progression.

Observational data revealed an inverse relationship between low vitamin D levels and HF morbidity and mortality. Dai et al. reported an inverse relationship between higher levels of serum vitamin D and all-cause mortality in a large observational study involving approximately 37.000 patients. The researchers also reported a cut-off value for serum level of vitamin D; in patients with levels below 50 nmol/L, a 10 nmol/L increase was associated with a 9% reduced risk of cardiovascular mortality, whereas in patients with levels above the cut-off point, there was no significant association between vitamin D concentrations and cardiovascular mortality [33]. Constanzo et al. analyzed the role of hypovitaminosis D in the prevalence of HF in a large cohort of adult Italians (no. 19.092) without prior HF events. Almost 562 hospital admissions due to HF were recorded over a median follow-up time of 6.2 years. In comparison to patients with normal 25-OHD, hypovitaminosis D, independent of other risk factors, was associated with a higher risk of hospitalization (HR: 1.61, 95%, CI: 1.06e2.43) and longer risk of total hospital days [30]. In a prospective cohort study derived from The Heart and Soul Study, almost 1000 patients with HF were followed-up on for an average of 8 years. During the study, 34.1% of patients experienced a cardiovascular event. The authors observed a link between patients with 25-OHD levels less than 20 ng/mL and cardiovascular events, with patients with 25-OHD levels less than 20 ng/mL having a 50% increased risk of cardiovascular events [27]. In a clinic-based study conducted by Gotsman et al., patients with HF (no. 3009) had lower serum vitamin D levels compared to the control group (no. 46 825), with a higher prevalence of vitamin D deficiency in HF patients (adjusted HR, 1.61 [95% CI: 1.08, 2.41]) [28]. Interestingly, in both groups, vitamin D deficiency was an independent predictor of reduced survival and higher mortality. However, the study’s design, with a higher proportion of patients in this study being female and the follow-up time being relatively low (518 days), can limit its applicability in a larger cohort of patients with cardiovascular diseases. In addition, the results from an observational study conducted by Liu. et al., on 13.131 patients with a medium follow-up time of 8 years, found a significant association between low levels of vitamin D and an increased risk of HF and premature death in patients aged ≥ 35 years [2]. Comparable results were reported in other small studies [28,31,32,52,53,54]. These results could imply that vitamin D could be a new risk factor for HF, and patients with HF and hypovitaminosis D should be monitored to ensure adequate vitamin D levels. However, despite the large number of patients included in these studies, the population analyzed was heterogeneous, with a wide age range and diverse ethnicities; thus, the results may not be generalizable to the larger population. 

Wang et al. published in 2022 a meta-analysis that found patients with low levels of 25-OHD had a nearly 35% higher risk of mortality compared to patients with normal or higher 25-OHD levels, suggesting that 25-OHD levels may be a significant predictor for mortality in HF patients. Furthermore, every 10 nmol/L decrease in 25-OHD levels was associated with a 10% increase in the risk of mortality [55]. Interestingly, contrary findings were discovered in another recent meta-analysis from 2022 that examined eight global observational studies with a total of 426.039 patients. The researchers discovered no link between vitamin D levels and the risk of myocardial infarction or the incidence of HF. The meta-analysis, on the other hand, found that patients with lower levels of vitamin D have a higher risk of major cardiac events (95% CI 1.24 to 2.98, *p* = 0.003; I2 = 90%) [56]. 

## 5. Vitamin D Deficiency and Supplementation 

Gotsman et al. tested vitamin D supplementation on 1783 HF patients with a standard dosage between 800-1000 units per day. In this study, vitamin D supplementation was associated with lower mortality in patients with vitamin D deficiency [28]. In contrast, a large study by Scragg et al. did not find any significant difference in the percentage of cardiovascular disease events between patients that received vitamin D supplementation versus the placebo group [57]. However, in this study, patients received a monthly dose of vitamin D versus a daily or a weekly one, which can be less effective. Moreover, only 24.9% of the population studied had vitamin D deficiency. Therefore, as suggested by other observational studies, patients with optimal vitamin D may not need vitamin D supplementation to prevent cardiovascular events (Table 2). 

Another double-blind placebo-controlled study, The VINDICATE Study, described that vitamin D supplementation with 4000 IU per day over the course of one year, on a cohort of 229 patients with HF, was associated with a significant improvement in cardiac function (6% higher ejection fraction- 95% confidence interval (CI):3.20 to 8.95; *p* < 0.0001]) and structure (reversal left ventricle remodeling with lower left ventricle end-diastolic [95% CI: −4.09 to −0.90; *p* Ľ 0.002] and end-systolic diameter [95% CI: −4.11 to −0.06 *p* Ľ 0.043]). However, this study failed to demonstrate their primary endpoint: they showed that vitamin D supplementation did not improve exercise capacity measured with the six-minute walk test [66]. Similar results were found in a small study by Boxer et al. after supplementation with a higher dosage of vitamin D (50.000 IU /week) [60]. Even though the six-minute walk test is usually used to assess the functional capacity of the patients, this test has great variability among patients; therefore, it is hard to predict its applicability. In contrast, in another recent study from 2022 conducted by Makoui et al. with the same dosage of vitamin D supplementation (50.000 IU/week), the authors found a significant improvement in heart failure class and cardiac function compared with the placebo group [60]. Increased ejection fraction of the left ventricle was also found in another small study conducted by Dalbeni et al. that found that vitamin D supplementation with the same dosage of 4000 IU/day for six months leads to an increased ejection fraction of the left ventricle in the treated group compared to control group (*p* = 0.007), with a significant positive correlation between vitamin D levels and ejection fraction (r Z 0.428; p Z 0.041) [61]. Similar results were found by Mohanty et al. in a recent non-randomized prospective study from 2022. After supplementation with a dosage of 60.000 IU vitamin D/week, patients had a significant decrease in NT-proBNP, with significant improvement in cardiac function and structure [65]. Interestingly, similar results were found in a study in infants with congestive HF and low levels of vitamin D. Despite different etiologies of HF, in association with HF therapy, after 3 months of vitamin D supplementation, significant improvement in cardiac structure and function was found (left ventricle ejection fraction, left ventricle end-diastolic and end-systolic diameter and myocardial performance index). In addition, after supplementation, higher levels of anti-inflammatory cytokines were found, with reduced concentrations of TNF-alpha and IL-6, known for their pro-inflammatory action (*p* < 0.001) [58]. 

Schroten et al. found a significant reduction in plasma renin levels after six weeks of vitamin D supplementation with 2000 IU in patients with HF [59]. Boxer et al. found that vitamin D supplementation with 50.000 IU/week caused a decrease in aldosterone serum levels after a follow-up of six months [62]. However, despite a higher dosage of vitamin D supplementation, they did not find clinical improvement or echocardiographic changes in patients with HF.

However, in the EVITA trial (Effect of Vitamin D on All-Cause mortality in Heart Failure Patients), one of the largest double-blinded trials, the results were different. With a 3-year follow-up and over 400 HF patients included, after vitamin D supplementation with 4.000 IU/day, results showed similar mortality between treated patients and the control group [63].

The results of large meta-analyses are controversial. On the one hand, three meta-analyses of vitamin D supplementation reported decreases in mortality. Neghedi et al., after including 11 trials, found that vitamin D supplementation can increase ejection fraction in HF patients by 3.3% (*p* = 0.006) [67]. Zhao et al. sustained that vitamin D supplementation could reduce left ventricle end-diastolic diameter (MD = −2.31 mm, 95% CI −4.15 to −0.47, *p* = 0.01) and increase left ventricle ejection fraction (MD = 4.18%, 95% CI 0.36 to 7.99, *p* = 0.03). However, those findings were more effective in patients with reduced ejection fraction [68]. A more recent and larger meta-analysis published in 2021 conducted by Kusunose et al. on 10.974 patients with chronic HF found that patients with vitamin D supplementation had lower in-hospital mortality [69]. 

On the other hand, another meta-analysis (2019) by Wang. et al. described no significant associations between vitamin D supplementation and mortality or left ventricular function. However, it found a significant association between vitamin D supplementation and improved quality of life and lower inflammatory response [70]. Similar results were found by Jiang et al. After the evaluation of eight clinical trials, the authors concluded that vitamin D supplementation did not influence left ventricle ejection fraction and NT-proBNP levels. However, in the vitamin D-treated group, inflammatory markers such as THF-α and C-reactive protein were significantly decreased [71]. 

Despite the conclusive evidence of the involvement of vitamin D in cardiovascular diseases, clinical trials that focused on vitamin D supplementation in adult patients with HF are controversial. Furthermore, in the pediatric population, there is a lack of randomized clinical trials that evaluate cardiovascular risk factors after vitamin D supplementation.

## 6. Discussions

Vitamin D appears to have a key role in cardiovascular health. Although many studies focused on the relationship between cardiovascular diseases and vitamin D, the results are still debatable. There are a few hypotheses regarding the contradictory results of the studies. First of all, these different results can be explained by the low number of patients included, with a short-term follow-up. Due to positive results obtained in experimental studies and in most observational studies, an increased time frame of vitamin D supplementation and a longer follow-up can change the results. Second, the heterogeneity of the patients included in most of the studies is significant with different ages or ethnicity. Third, most studies used 25-OHD levels to describe vitamin D deficiency, not the biologically active form. However, the results were similar in the few studies that analyzed the active form of vitamin D, but it is important to mention that due to numerous factors that can influence the active form of vitamin D, for accurate clinical research, it is insufficient to determine the active metabolite in isolation. The results should be interpreted in association with calcium and phosphorus concentration, PTH values or if renal and bone mineral diseases are present. In addition, there is no consensus regarding hypovitaminosis D in terms of normal levels. Due to the lack of standardization regarding vitamin D levels and measurements, the comparison between different studies is still a problem. Some studies considered hypovitaminosis D when serum levels were below 30 ng/mL, while others considered values below 25 ng/mL. Another critical issue is that sunlight exposure and different daily intakes of vitamin D between patients can interfere with the results. Therefore, a single measurement of vitamin D levels used in most observational studies may not be reliable. Additionally, vitamin D deficiency is usually found in association with other HF risk factors and therefore is difficult to assess if hypovitaminosis D is a cause or a consequence of HF. 

Furthermore, the clinical trials that analyzed vitamin D supplementation are contradictory and without conclusive data that can support vitamin D supplementation. Moreover, some studies employed a higher dosage than that recommended by different societies. Because there is no consensus regarding the recommended dosage of vitamin D, different dosages of vitamin D may produce different results. It should be taken into account that vitamin D supplementation may be beneficial in vitamin D-deficient patients with an effect that may be dose-dependent. 

Therefore, we consider that vitamin D should be taken into consideration as a potential, easily modifiable cardiovascular marker due to the positive effects demonstrated in most experimental and observational studies, meta-analyses and controlled trials. Further studies should conduct serial analyses of vitamin D levels to better understand the role of vitamin D as a cardiovascular risk in the long term. In addition, due to its low risk of side effects and low-cost, vitamin D supplementation could be a viable strategy in HF therapy. More well-designed standardized randomized trials are needed to determine whether vitamin D supplementation can benefit patients with HF.

## 7. Conclusions

Cardiovascular diseases continue to be a major public health concern worldwide. Overall, even though most experimental and observational studies on the relationship between vitamin D and HF in both adult and pediatric populations have yielded positive results, the potential mechanism by which vitamin D is involved in HF pathogeny is still poorly understood. Furthermore, the evidence to support vitamin D supplementation in HF patients is still insufficient due to the lack of randomized control trials. Vitamin D, on the other hand, remains a promising low-cost modifiable cardiovascular marker, and larger studies are needed to investigate this association with specific populations and reach a consensus regarding vitamin D dosage. 

## Figures and Tables

**Table 1:** Major studies regarding the clinical relevance of vitamin D in HF adult patients.

First Author/Year	Study Design	Sample Size/Age	Mean Follow-Up	Outcome/Endpoint	Results
Liu et al., 2012 [2]	Prospective observational study	13.131 ≥35 years	8 years	Cardiovascular events and mortality	1.11 HR (95% CI 0.93–1.33) and 1.40 HR (95% CI 1.17–1.68) for all-cause mortality in those with serum 25(OH)D insufficiency and those with deficiency, respectively, compared to those with normal serum 25(OH)D level
Welles et al., 2013 [27]	Prospective observational study	946 >18 years	8 years	Cardiovascular events	1.50 HR (95% CI 1.19–1.90) for cardiovascular events in patients with vitamin D deficiency (<20 nmol/L).
Gotsman et al., 2014 [28]	Retrospective Case-control	3009 75.9 ± 10.7 years	518 days	Cardiovascular mortality	1.52 HR (95% CI 1.21–1.92) for 1.6-year mortality in patients with vitamin D deficiency (<25 nmol/L).
Grunson et al., 2015 [29]	Prospective observational study	170 n = 134 males	4.1 years	Cardiovascular mortality	1,25(OH)2D significantly predicted long-term CV death in HF patients and heart transplantation
Constanzo et al., 2017 [30]	Prospective observational study	19.092 35–99 years	6.2 years	HF risk of hospitalization	1.61 HR (95% CI 1.06–2.43) in patients with vitamin D deficiency (<10 nmol/L), 1.14 HR (95% CI 0.81–1.61) in patients with vitamin D insufficiency (10–29 nmol/L) compared with those with a normal level.
Saponaro et al., 2018 [31]	Prospective observational study	247 65 ± 13 years	2.10 years	Cardiovascular mortality	0.62 (95% CI 0.39–0.99) 25-OHD levels as an independent predictor of HF related-death Vitamin D insufficiency was associated with reduced survival in HF patients (log-rank *p* = 0.017).
Nolte et al., 2019 [32]	Prospective observational study	787 50–85 years	5 years	Cardiovascular events, mortality and hospitalization	Lower 25(OH)D levels (per 10 ng/mL decrease) - tended to be associated with higher 5-year mortality- 1.55 HR [*p* = 0.05, 1.00; 2.42]. - related to an increased rate of cardiovascular hospitalizations- 1.74 HR [*p* = 0.02, 1.08; 2.80], and remained
Dai et al., 2021 [33]	Prospective observational study	37.079 40–69 years	11.7 years	Cardiovascular mortality	Increasing levels in serum 25(OH)D were independently associated with a decreased risk of all-cause and cause-specific mortality and then reached a plateau at around 50 nmol/L 25(OH)D

HR: hazard ratio, 25-OHD: 25-dihydroxivitamin D, HF: heart failure, CVD: cardiovascular death.

**Table 2:** Major randomized-controlled trials regarding Vitamin D supplementation in HF patients.

First Author/Year	Study Design	Inclusion Criteria	Sample Size/Age		Vitamin D Dosage and Duration of Treatment	Follow-Up	Outcome
			Treatment	Placebo			
Shedeed et al., 2012 [58]	Prospective double-blinded	Chronic HF patients < 18 years, LVEF < 40%	42 patients 10.3 ± 4.6 years	38 patients 11.2 ± 3.5 years	1.000 IU/day	3 months	Inflammatory markers, Echocardiography parameters
Schroten et al., 2013 [59]	Prospective double-blinded	Chronic HF patients ≥ 18 years	50 patients 64 ± 10 years	51 patients 63.5 ± 11.1 years	2.000 IU/day	6 weeks	Laboratory, urine analysis
Boxer et al., 2013 [60]	Prospective double-blinded	Chronic HF patients ≥ 50 years, NYHA II-IV, 25-OHD < 37.5 ng/ml	30 patients 65.8 ± 10.6 years	30 patients 66.0 ± 10.4 years	50.000 IU/week	6 months	Peak Oxygen uptake, 6MWT
Dalbeni et al., 2013 [61]	Prospective double-blinded	Chronic HF, Patients >40 years, LVEF < 55%, NYHA > II, 25-OHD < 30 ng/ml	13 patients. 74.2 (66.6–81.9) years	10 patients 74.3 (62.7–85) years	4.000 IU/day	6 months	Inflammatory markers, Echocardiography parameters
Boxer et al., 2014 [62]	Prospective double-blinded	Chronic HF patients, NYHA II-IV, 25-OHD < 37.5 ng/ml	Mean age 65.8 ± 10.6 years	Mean age 66.0 ± 10.4 years	50.000 IU/week	6 months	Laboratory, urine analysis, Echocardiography parameters
Moretti et al., 2017	Prospective double-blinded	Chronic HF patients ≥ 18 years, NYHA II-III	17 patients Mean age 67 ± 11 years	19 patients Mean age 65 ± 16 years	10.000 IU/day	6 months	Quality of life, Laboratory, and Cardiopulmonary functions
Scragg et al., 2017 [57]	Prospective double-blinded	Age 50–84 years	2558 patients	2552 patients	200.000 IU first month 100.000 IU/monthly	3.3 years	CVD mortality
Zitterman et al., 2019 [63]	Prospective double-blinded	25-OHD < 75 ng/ml	80 patients Mean age 55.5 (48–61) years	81 patients Mean age 54 (46–58) years	4.000 IU/day	3 years	CVD mortality, HF hospitalization, listing for heart transplantation, hypercalcemia
Makoui et al., 2020 [64]	Prospective double-blinded	Chronic HF patients, Patients > 40 years, NYHA I-III	41 patients 61.68 ± 19.8 years	41 patients 62.12 ± 18.2 years	50.000 IU/week	2 months	Echocardiography parameters
Mohanty et al., 2022 [65]	Prospective non-randomized	Chronic HF patients Patients >18 years	97 patients 53.77 ± 13.02 years	-	60.000 IU/week	3 months	Laboratory, Echocardiography parameters

HF- heart failure, LVEF- left ventricle ejection fraction, CVD- cardiovascular diseases, NYHA- New York Heart Association, 25-OHD- 25-dihydroxy vitamin D, IU- international units

## Data Availability

Not applicable.

## References

[1] Ponikowski P., Voors A.A., Anker S.D., Bueno H., Cleland J.G., Coats A.J., Falk V., González-Juanatey J.R., Harjola V.P., Jankowska E.A. (2016). Authors/Task Force M, Document R. 2016 ESC Guidelines for the diagnosis and treatment of acute and chronic heart failure: The Task Force for the diagnosis and treatment of acute and chronic heart failure of the European Society of Cardiology (ESC). Developed with the special contribution of the Heart Failure Association (HFA) of the ESC. Eur. J. Heart Fail..

[2] Liu L., Chen M., Hankins S.R., Nùñez A.E., Watson R.A., Weinstock P.J., Newschaffer C.J., Eisen H.J. (2012). Serum 25-Hydroxyvitamin D Concentration and Mortality from Heart Failure and Cardi-ovascular Disease, and Premature Mortality from All-Cause in United States Adults. Am. J. Cardiol..

[3] Zittermann A., Trummer C., Theiler-Schwetz V., Lerchbaum E., März W., Pilz S. (2021). Vitamin D and Cardiovascular Disease: An Updated Narrative Review. Int. J. Mol. Sci..

[4] Zehnder D., Bland R., Chana R.S., Wheeler D.C., Howie A.J., Williams M.C., Stewart P.M., Hewison M. (2002). Synthesis of 1,25-Dihydroxyvitamin D_3_ by Human Endothelial Cells Is Regulated by Inflammatory Cytokines: A Novel Autocrine Determinant of Vascular Cell Adhesion. J. Am. Soc. Nephrol..

[5] Chen M., Yu L., Liu Q., Jiang H., Zhou S. (2015). Vitamin D: A potential important therapeutic target for atrial fibrillation. Int. J. Cardiol..

[6] Mandarino N.R., Júnior F.D.C.M., Salgado J.V.L., Lages J.S., Filho N.S. (2015). Is Vitamin D Deficiency a New Risk Factor for Cardiovascular Disease?. Open Cardiovasc. Med. J..

[7] Gunta S.S., Thadhani R.I., Mak R.H. (2013). The effect of vitamin D status on risk factors for cardiovascular disease. Nat. Rev. Nephrol..

[8] D’Amore C., Marsico F., Parente A., Paolillo S., De Martino F., Gargiulo P., Ferrazzano F., De Roberto A., La Mura L., Marciano C. (2017). Vitamin D deficiency and clinical outcome in patients with chronic heart failure: A review. Nutr. Metab. Cardiovasc. Dis..

[9] Misra M., Pacaud D., Petryk A., Collett-Solberg P.F., Kappy M., on behalf of the Drug and Therapeutics Committee of the Lawson Wilkins Pediatric Endocrine Society (2008). Vitamin D Deficiency in Children and Its Management: Review of Current Knowledge and Recommendations. Pediatrics.

[10] Holick M.F., Binkley N.C., Bischoff-Ferrari H.A., Gordon C.M., Hanley D.A., Heaney R.P., Murad M.H., Weaver C.M. (2011). Evaluation, Treatment, and Prevention of Vitamin D Deficiency: An Endocrine Society Clinical Practice Guideline. Med. J. Clin. Endocrinol. Metab..

[11] Institute of Medicine (2011). Dietary Reference Intakes: Calcium and Vitamin D.

[12] EFSA NDA Panel (EFSA Panel on Dietetic Products, Nutrition and Allergies) (2016). Scientific opinion on dietary reference values for vitamin D.. EFSA J..

[13] DGE (German Nutrition Society), Österreichische Gesellschaft für Ernährung, Schweizerische Gesellschaft für Ernährungs-forschung, Schweizerische Vereinigung für Ernährung (2012). D-A-CH Referenzwerte für die Nährstoffzufuhr.

[14] Gezmish O., Black M.J. (2013). Vitamin D Deficiency in Early Life and the Potential Programming of Cardiovascular Disease in Adulthood. J. Cardiovasc. Transl. Res..

[15] Hollis B.W., Wagner C.L. (2004). Vitamin D requirements during lactation: High-dose maternal supplementation as therapy to prevent hypovitaminosis D for both the mother and the nursing infant. Am. J. Clin. Nutr..

[16] Kim D.H., Sabour S., Sagar U.N., Adams S., Whellan D.J. (2008). Prevalence of Hypovitaminosis D in Cardiovascular Diseases (from the National Health and Nutrition Examination Survey 2001 to 2004). Am. J. Cardiol..

[17] Beveridge L.A., Witham M.D. (2013). Vitamin D and the cardiovascular system. Osteoporos. Int..

[18] Simpson R.U., Hershey S.H., Nibbelink K.A. (2007). Characterization of heart size and blood pressure in the vitamin D receptor knockout mouse. J. Steroid Biochem. Mol. Biol..

[19] Li Y.C., Kong J., Wei M., Chen Z.-F., Liu S.Q., Cao L.-P. (2002). 1,25-Dihydroxyvitamin D3 is a negative endocrine regulator of the renin-angiotensin system. J. Clin. Investig..

[20] Charytan D.M., Padera R.F., Helfand A.M., Zeisberg E.M. (2015). Association of activated vitamin D use with myocardial fibrosis and capillary supply: Results of an autopsy study. Ren. Fail..

[21] Meredith A.J., McManus B.M. (2013). Vitamin D in Heart Failure. J. Card. Fail..

[22] Shaheen M., Cheema Y., Shahbaz A.U., Bhattacharya S.K., Weber K.T. (2011). Intracellular calcium overloading and oxidative stress in cardiomyocyte necrosis via a mitochondriocentric signal-transducer-effector pathway. Exp. Clin. Cardiol..

[23] Weber K.T., Weglicki W.B., Simpson R.U. (2008). Macro- and micronutrient dyshomeostasis in the adverse structural remodelling of myocardium. Cardiovasc. Res..

[24] Anderson J.L., Vanwoerkom R.C., Horne B.D., Bair T.L., May H.T., Lappé D.L., Muhlestein J.B. (2011). Parathyroid hormone, vitamin D, renal dysfunction, and cardiovascular disease: Dependent or independent risk factors?. Am. Heart J..

[25] Wannamethee S.G., Welsh P., Papacosta O., Lennon L., Whincup P.H., Sattar N. (2014). Elevated Parathyroid Hormone, But Not Vitamin D Deficiency, Is Associated With Increased Risk of Heart Failure in Older Men With and Without Cardiovascular Disease. Circ. Heart Fail..

[26] Kolaszko A., Nowalany-Kozielska E., Ceranowicz P., Morawiec B., Kubiak G. (2018). The Role of Parathyroid Hormone and Vitamin D Serum Concentrations in Patients with Cardiovascular Diseases. Dis. Markers.

[27] Laranjo S., Trigo C., Pinto F.F. (2014). Dual etiology of dilated cardiomyopathy: The synergistic role of Vitamin D deficiency. Rev Port Cardiol..

[28] Raafat D.M., El-Asheer O.M., Mahmoud A.A., Darwish M.M., Osman N.S. (2021). Vitamin D Status in Children with Idiopathic Dilated Cardiomyopathy. J. Child Sci..

[29] Otani K., Higa Y., Tanaka K., Adachi H., Nakazono A., Nabeshima Y., Honda M., Otsuji Y., Takeuchi M. (2018). Relations of Vitamin D Status With B-Type Natriuretic Peptide Levels and the Risk of Cardiac Events in Japanese Subjects With Heart Failure. J. Card. Fail..

[30] Fall T., Shiue I., Geijerstam P.B.A., Sundström J., Ärnlöv J., Larsson A., Melhus H., Lind L., Ingelsson E. (2012). Relations of circulating vitamin D concentrations with left ventricular geometry and function. Eur. J. Hear. Fail..

[31] Welles C.C., Whooley M.A., Karumanchi S.A., Hod T., Thadhani R., Berg A.H., Ix J.H., Mukamal K.J. (2014). Vitamin D deficiency and cardiovascular events in patients with coronary heart disease: Data from the Heart and Soul Study. Am. J. Epidemiol..

[32] Gotsman I., Shauer A., Zwas D.R., Hellman Y., Keren A., Lotan C., Admon D. (2012). Vitamin D deficiency is a predictor of reduced survival in patients with heart failure; vitamin D supplementation improves outcome. Eur. J. Heart Fail..

[33] Ameri P., Canepa M., Milaneschi Y., Spallarossa P., Leoncini G., Giallauria F., Strait J.B., Lakatta E.G., Brunelli C., Murialdo G. (2012). Relationship between vitamin D status and left ventricular geometry in a healthy population: Results from the Baltimore Longitudinal Study of Aging. J. Intern. Med..

[34] Gruson D., Ferracin B., Ahn S.A., Zierold C., Blocki F., Hawkins D.M., Bonelli F., Rousseau M.F. (2015). 1,25-Dihydroxyvitamin D to PTH(1–84) Ratios Strongly Predict Cardiovascular Death in Heart Failure. PLoS ONE.

[35] Costanzo S., De Curtis A., Di Castelnuovo A., Persichillo M., Bonaccio M., Pounis G., Cerletti C., Donati M., de Gaetano G., Iacoviello L. (2018). Serum vitamin D deficiency and risk of hospitalization for heart failure: Prospective results from the Moli-sani study. Nutr. Metab. Cardiovasc. Dis..

[36] Saponaro F., Saba A., Frascarelli S., Prontera C., Clerico A., Scalese M., Sessa M.R., Cetani F., Borsari S., Pardi E. (2018). Vitamin D measurement and effect on outcome in a cohort of patients with heart failure. Endocr. Connect..

[37] Nolte K., Herrmann-Lingen C., Platschek L., Holzendorf V., Pilz S., Tomaschitz A., Düngen H., Angermann C.E., Hasenfuß G., Pieske B. (2019). Vitamin D deficiency in patients with diastolic dysfunction or heart failure with preserved ejection fraction. ESC Hear. Fail..

[38] Dai L., Liu M., Chen L. (2021). Association of Serum 25-Hydroxyvitamin D Concentrations With All-Cause and Cause-Specific Mortality Among Adult Patients With Existing Cardiovascular Disease. Front. Nutr..

[39] Seferović P.M., Polovina M., Bauersachs J., Arad M., Ben Gal T., Lund L.H., Felix S.B., Arbustini E., Caforio A.L.P., Farmakis D. (2019). Heart failure in cardiomyopathies: A position paper from the Heart Failure Association of the European Society of Cardiology. Eur. J. Heart Fail..

[40] Karoli R., Priya S., Siddiqi Z., Fatima J., Gupta S., Mishra R. (2016). Study of vitamin D status in patients with dilated cardiomyopathy at a teaching hospital in North India. J. Cardiovasc. Echography.

[41] Polat V., Bozcali E., Uygun T., Opan S., Karakaya O. (2015). Low vitamin D status associated with dilated cardiomyopathy. Int. J. Clin. Exp. Med..

[42] Rahman MA, Galal H and Salem Omar AM. Correlation between serum Vitamin D level and cardiac function: Echocardio-graphic assessment. Egypt. Heart J. 2015, 67:299-305.

[43] Pandit A., Mookadam F., Boddu S., Pandit A.A., Tandar A., Chaliki H., Cha S., Lee H.R. (2014). Vitamin D levels and left ventricular diastolic function. Open Hear..

[44] Elidrissy A.T., Munawarah M., Alharbi K.M. (2012). Hypocalcemic rachitic cardiomyopathy in infants. J. Saudi Heart Assoc..

[45] Karabayir N., Aktas D. (2016). Dilated cardiomyopathy due to Vitamin D deficiency. Int. J. Clin. Cardiol..

[46] Thomas G.N., Hartaigh B., Bosch J.A., Pilz S., Loerbroks A., Kleber M.E., Fischer J.E., Grammer T.B., Böhm B.O., März W. (2012). Vitamin D Levels Predict All-Cause and Cardiovascular Disease Mortality in Subjects With the Metabolic Syndrome: The Ludwigshafen Risk and Cardiovascular Health (LURIC) Study. Diabetes Care.

[47] Parikh S., Guo D.-H., Pollock N.K., Petty K., Bhagatwala J., Gutin B., Houk C., Zhu H., Dong Y. (2012). Circulating 25-Hydroxyvitamin D Concentrations Are Correlated With Cardiometabolic Risk Among American Black and White Adolescents Living in a Year-Round Sunny Climate. Diabetes Care.

[48] Sena-Evangelista K.C.M., Dantas-Komatsu R.C.S., Freire F.L.d.A., de Lira N.R.D., Diniz R.V.Z., Lima S.C.V.C., Pedrosa L.F.C. (2021). Vitamin D status and predictors of 25-hydroxyvitamin D levels in patients with heart failure living in a sunny region. Nutr. Hosp. Organo Of. Soc. Española Nutr. Parenter. Y Enter..

[49] Ma Y.-H., Zhou Y.-L., Yue C.-Y., Zhang G.-H., Deng L., Xie G.-H., Xu W.-P., Shen L.-S. (2015). Vitamin D Deficiency Contributes to the Reduction and Impaired Function of NaïveCD45RA+Regulatory T Cell in Chronic Heart Failure. J. Immunol. Res..

[50] Afifeh A.M.S., Verdoia M., Nardin M., Negro F., Viglione F., Rolla R., De Luca G. (2021). Determinants of vitamin D activation in patients with acute coronary syndromes and its correlation with inflammatory markers. Nutr. Metab. Cardiovasc. Dis..

[51] Roffe-Vazquez D.N., Huerta-Delgado A.S., Castillo E.C., Villarreal-Calderón J.R., Gonzalez-Gil A.M., Enriquez C., Garcia-Rivas G., Elizondo-Montemayor L. (2019). Correlation of Vitamin D with Inflammatory Cytokines, Atherosclerotic Parameters, and Lifestyle Factors in the Setting of Heart Failure: A 12-Month Follow-Up Study. Int. J. Mol. Sci..

[52] Belen E., Sungur A., Sungur M.A. (2015). Vitamin D levels predict hospitalization and mortality in patients with heart failure. Scand. Cardiovasc. J..

[53] Cubbon R.M., Lowry J.E., Drozd M., Hall M., Gierula J., Paton M.F., Byrom R., Kearney L.C., Barth J.H., Kearney M.T. (2018). Vitamin D deficiency is an independent predictor of mortality in patients with chronic heart failure. Eur. J. Nutr..

[54] Perge P., Boros A.M., Gellér L., Osztheimer I., Szilágyi S., Tahin T., Apor A., Nagy K.V., Zima E., Molnár L. (2019). Vitamin D Deficiency Predicts Poor Clinical Outcomes in Heart Failure Patients Undergoing Cardiac Resynchronization Therapy. Dis. Markers.

[55] Wang X.M., Wang J.M., Gao T.M., Sun H.M., Yang B. (2022). Is vitamin D deficiency a risk factor for all-cause mortality and rehospitalization in heart failure patients?: A systematic review and meta-analysis. Medicine.

[56] Jaiswal V., Ishak A., Ang S.P., Pokhrel N.B., Shama N., Lnu K., Varghese J.S., Storozhenko T., Chia J.E., Naz S. (2022). Hypovitaminosis D and cardiovascular outcomes: A systematic review and meta-analysis. IJC Hear. Vasc..

[57] Scragg R., Stewart A.W., Waayer D., Lawes C.M.M., Toop L., Sluyter J., Murphy J., Khaw K.-T., Camargo C.A. (2017). Effect of Monthly High-Dose Vitamin D Supplementation on Cardiovascular Disease in the Vitamin D Assessment Study. JAMA Cardiol..

[58] Shedeed S.A. (2012). Vitamin D Supplementation in Infants With Chronic Congestive Heart Failure. Pediatr. Cardiol..

[59] Schroten N.F., Ruifrok W.P., Kleijn L., Dokter M.M., Silljé H.H., Heerspink H.J.L., Bakker S.J., Kema I.P., van Gilst W., van Veldhuisen D.J. (2013). Short-term vitamin D3 supplementation lowers plasma renin activity in patients with stable chronic heart failure: An open-label, blinded end point, randomized prospective trial (VitD-CHF trial). Am. Heart J..

[60] Boxer R.S., Kenny A.M., Schmotzer B.J., Vest M., Fiutem J.J., Piña I.L. (2013). A Randomized Controlled Trial of High-Dose Vitamin D3 in Patients With Heart Failure. JACC Heart Fail..

[61] Dalbeni A., Scaturro G., Degan M., Minuz P., Delva P. (2014). Effects of six months of vitamin D supplementation in patients with heart failure: A randomized double-blind controlled trial. Nutr. Metab. Cardiovasc. Dis..

[62] Boxer R.S., Hoit B.D., Schmotzer B.J., Stefano G., Gomes A., Negrea L. (2014). The Effect of Vitamin D on Aldosterone and Health Status in Patients With Heart Failure. J. Card. Fail..

[63] Zittermann A., Ernst J.B., Prokop S., Fuchs U., Dreier J., Kuhn J., Knabbe C., Börgermann J., Berthold H.K., Pilz S. (2018). Daily Supplementation with 4000 IU Vitamin D3 for Three Years Does Not Modify Cardiovascular Risk Markers in Patients with Advanced Heart Failure: The Effect of Vitamin D on Mortality in Heart Failure Trial. Ann. Nutr. Metab..

[64] Hassanzadeh-Makoui R., Jamei M., Hassanzadeh-Makoui M., Khederlou H. (2020). Effects of Vitamin D on Left Ventricular Ejection Fraction in Patients with Systolic Heart Failure: A Double-Blind Randomized Clinical Trial. Int. J. Endocrinol. Metab..

[65] Mohanty V., Pathania M., Bhasi A. (2022). Effect of vitamin supplementation in patients of congestive heart failure deficient in vitamin D: A study at a tertiary care center of North India. Ann. Afr. Med..

[66] Witte K.K., Byrom R., Gierula J., Paton M.F., Jamil H.A., Lowry J.E., Gillott R.G., Barnes S.A., Chumun H., Kearney L.C. (2016). Effects of Vitamin D on Cardiac Function in Patients With Chronic HF: The VINDICATE Study. J. Am. Coll. Cardiol..

[67] Naghedi A., Haghaninejad H., Varastehravan H., Naghedi A., Farshadi N. (2021). Effect of vitamin D supplements on left ventricular ejection fraction in patients with heart failure: A systematic review and meta-analysis of randomized controlled trials. Rev. Port. de Cardiol..

[68] Zhao J.-D., Jia J.-J., Dong P.-S., Zhao D., Yang X.-M., Li D.-L., Zhang H.-F. (2018). Effect of vitamin D on ventricular remodelling in heart failure: A meta-analysis of randomised controlled trials. BMJ Open.

[69] Kusunose K., Okushi Y., Okayama Y., Zheng R., Abe M., Nakai M., Sumita Y., Ise T., Tobiume T., Yamaguchi K. (2021). Association between Vitamin D and Heart Failure Mortality in 10,974 Hospitalized Individuals. Nutrients.

[70] Wang T., Liu Z., Fu J., Min Z. (2019). Meta-analysis of vitamin D supplementation in the treatment of chronic heart failure. Scand. Cardiovasc. J..

[71] Jiang W.-L., Gu H.-B., Zhang Y.-F., Xia Q., Qi J., Chen J.-C. (2015). Vitamin D Supplementation in the Treatment of Chronic Heart Failure: A Meta-analysis of Randomized Controlled Trials. Clin. Cardiol..

